# Defining Vitality Using Physical and Mental Well-Being Measures in Nursing Homes: A Prospective Study

**DOI:** 10.1007/s12603-019-1285-8

**Published:** 2019-10-19

**Authors:** E. Masciocchi, Mathieu Maltais, K. El Haddad, K. Virecoulon Giudici, Y. Rolland, B. Vellas, P. de Souto Barreto

**Affiliations:** 1 Gérontopôle de Toulouse, Institut du Vieillissement, Bâtiment B, Centre Hospitalo-Universitaire de Toulouse, 37 allée Jules Guesdes, 31000 Toulouse, France; 2 Division of Geriatrics, University of Verona, Verona, Italy; 3 UMR INSERM, 1027, University of Toulouse III, Toulouse, France Faculté de médecine, 37 allées Jules Guesde, 31000 Toulouse, France

**Keywords:** Hand grip strength, mental vitality, nursing homes, vitality

## Abstract

**Objectives:**

To propose an objective definition of vitality and to evaluate its predictive value regarding the evolution of functional ability, as well as the risk of hospitalization and mortality in very old NH residents.

**Design:**

Observational study.

**Settings:**

Nursing homes.

**Participants:**

541 participants.

**Measurements:**

We operationalized tree definitions of vitality (binary variables discriminating vital from non-vital individuals): Mental vitality, assessed using three items of the geriatric depression scale; Physical vitality measured through hand grip strength test; and combined vitality, which combined mental and physical vitality definitions. Outcome measures were the 1-year evolution of functional ability as measured by a scale of activities of daily living (ADL) (score from 0 to 6) and the incidence of hospitalizations and mortality (time-to-event).

**Results:**

First, 204 (37.7%) residents were defined as mentally vital. Second, 139 (27.5%) residents were defined as physically vital. And 52 (9.6%) were defined as vital when combining physical and. Combined vitality was associated with a reduced risk of hospitalization compared to combined non-vitality. Physically vital residents were associated with a reduced risk of mortality. No prospective associations were found between vital and non-vital individuals on the evolution of ADL scores across the three vitality definitions. But mentally vital individuals were associated with a worsening of ADL score.

**Conclusions:**

Better combined vitality seems to be associated with a reduced risk for hospitalizations, but more studies are needed to confirm a valid measurement of vitality in people living in NH in regards to ADL and mortality.

**Electronic Supplementary Material:**

Supplementary material is available for this article at 10.1007/s12603-019-1285-8 and is accessible for authorized users.

## Introduction

Vitality is one of the five intrinsic capacity (IC) domains defined by the World Health Organization (WHO) and that strongly contribute to a healthy aging (1, 2). Despite its importance for determining the health trajectories during aging, no consensus exists about the best operational definition of vitality, in particular among vulnerable older adults living in nursing homes (NH).

According to the Oxford English Dictionary, vitality is defined as “the state of being strong and active; energy” (3). In previous studies, the concept of vitality was often connected to that of psychological energy, aliveness, meaning in life and self-esteem (4–6). One of the most widely used questionnaires to assess health-related quality of life, the 36-Item Short Form Survey (SF-36) (7), evaluates vitality using four subjective questions about feelings of energy, with limited usability among NH residents (8). Previous researches have also recognized the importance of a physical component for assessing vitality (9, 10). To the best of our knowledge, the best operational definition of vitality able to predict clinical outcomes in NH residents has never been determined or even investigated. Furthermore, whether vitality should be defined as a psychological feeling of energy, a physical test or a combination of both remains to be established. From a clinical standpoint, having an easy assessment of vitality could facilitate the detection of people at risk of adverse outcomes in NH residents.

The purpose of this study was to investigate the predictive value of three different operational definitions of vitality (ie, mental vitality, physical vitality, or a combination of both) on functional ability, hospitalization and mortality in NH residents using data from a 1-year observational study.

## Methods

We performed secondary analysis from the “Incidence of pNeumonia and related ConseqUences in nursing home Resident” (INCUR) study (11). Concisely, the INCUR study was a prospective observational study aimed at evaluating the incidence of pneumonia events in institutionalized people over a period of 12 months. The study protocol and more detailed information on the methods have been previously described (11). The study was conducted in 13 nursing homes in southwestern France and involved 800 participants. Inclusion criteria were 1) to be 60 or more years old and 2) to present an activity daily living (ADL) score between 2 and 5 (the French GIR score, which varies from 1 to 6, with 6 representing a fully independent individual (12)). Exclusion criteria were 1) to live in the NH for less than 30 days at the moment of baseline data collection and 2) refusal to participate at the study by the patient or his/her family.

The study protocol was approved by the Ethical Committee of the Centre Hospitalier Universitaire de Toulouse which waived the need for a participants’ signed consent because this observational study made part of current clinical practices. All participants and/or proxies were informed about the research activity and left free to accept or refuse their participation.

### Participants

From the 800 participants recruited, 541 had the required data to operationalize vitality. The average age was 88 years old (83–91) and 72% of the sample were women. Sociodemographic data was collected from the medical records of the patients. Eligible participants received three clinical evaluations (at baseline, 6 and 12 months) during the follow-up.

### Vitality

We defined mental vitality using the following three questions of the 10-item Geriatric Depression Scale (GDS) (13): 1) Are you basically satisfied with your life? (YES=0, NO=1); 2) Do you feel that your life is empty? (NO=0, YES=1); 3) Do you feel full of energy? (YES=0, NO=1). A total score of zero defined people with high vitality, whereas a score between one and three defined people with low vitality. Physical vitality was assessed using the hand grip strength test which is a vital sign and a marker of physical energy (14). Hand grip strength (HGS) was measured twice with each hand using a hand-held dynamometer (model Jamar) and the average of the best results obtained with both hands has been used for the analyses. The threshold of HGS was defined as the 75th percentile (15) among males and females separately (physical vitality = HGS ≥23 kg in males and ≥ 13.5 kg in females). Combined vitality (very low or very high) was defined as meeting vitality criteria for both mental and physical vitality.

### Outcome Measures

Three outcomes were evaluated: 1-year evolution of ADL (i.e. bathing, dressing, toileting, transferring, continence, and feeding) scores, which varies from six (i.e. independent) to zero (i.e. completely dependent) (16). It was assessed at baseline, 6 months and 12 months. The 1-year risk of hospitalizations (yes/no) and mortality were investigated using the date to the first event for hospitalization and the date of death for mortality.

### Confounders

All models were adjusted for the following potential confounders: age, sex, duration of institutionalization, instrumental activity of daily living (IADL) (17), short physical performance battery (SPPB) score (18), mini nutritional assessment (MNA) (19) and the Charlson comorbidity index (20).

### Statistical analysis

Descriptive statistics are presented as median and interquartile ranges, absolute numbers and percentages as appropriate. Baseline comparisons between individuals with high or low vitality were tested using the chi-square test and the Wilcoxon rank sum test, as appropriate. A linear mixed-effect regression model adjusted for confounders was used for analyzing the evolution of ADL score between high and low vitality. Adjusted means for each group were obtained from the models and between-group adjusted mean differences were calculated for each time point. Sensitivity analyses were performed by removing individuals diagnosed with dementia and/or depression (n=279). For the incidence of hospitalization and mortality, Kaplan-Meier graphs and Cox proportional hazard models adjusted for confounders were run. For participants who developed the event, we calculated time to event in months using time to first hospitalization and time to date of death for mortality. Participants not experiencing the event were censored at their follow-up time. Statistical significance was determined as p<0.05. Analyses were performed using Stata version 14.0 (StataCorp, College Station, Texas).

## Results

The characteristics of the population at baseline according to their vitality status (high vs. low vitality) across vitality definitions are shown in table 1. For the 541 participants, 204 (37.7%), 139 (25.7%) and 52 (9.6%) were rated as individuals with high vitality according to mental, physical and combined definitions, respectively. At baseline, compared to low vitality, subjects with high vitality in all of the three definitions operationalized were significantly younger, had lower depressive symptoms, better physical performance and higher ADL scores.

**Table 1:** Baseline characteristics of long-term care institution residents according to mental, physical and combined vitality definitions

	**Total n = 541**	**Mental Vitality’**		**p**	**Physical Vitality”**		**p**	**Combined Vitality’”**		**p**
		**High vitality n = 204**	**Low vitality n =337**		**High vitality n = 139**	**Low vitality n = 402**		**High vitality n = 52**	**Low vitality n = 489**	
Age	88 [83–91]	87 [82–91]	88 [83–91]	0.05	86 [81–90]	88 [83–92]	<0.001	86 [82–88.5]	88 [83–91]	0.009
Sex, female (%)	390 (72.0)	139 (68.1)	251 (74.4)	0.1	101 (72.6)	289 (71.8)	0.8	40 (76.9)	350 (71.5)	0.4
Duration of Institutionalization (years)	2.0 [0.8–4.5]	2.3 [0.9–5.6]	1.9 [0.7–4.0]	0.02	2.1 [0.7–4.9]	2.0 [0.8–4.4]	0.7	2.0 [0.6–5.6]	2.1 [0.8–4.4]	0.9
GDS score (/10)*	2 [1–4]	1 [0–1]	4 [2–5]	<0.001	2 [1–4]	2 [1–5]	0.7	1 [0–2]	2 [1–5]	<0.001
ADL score (/6)°	3 [1–4]	3 [2–5]	2 [1–4]	<0.001	3 [2–5]	2 [1–4]	<0.001	4 [2.5–5]	3 [1–4]	0.002
IADL score (/4)†	1 [0–1]	1 [0–1]	1 [0–1]	0.007	1 [1–1]	1 [0–1]	<0.001	1 [0.5–1]	1 [0–1]	0.002
BMI (kg/m2)***	25.3 [22.1–28.5]	25.4 [21.9–28.8]	25.2 [22.2–28.5]	0.9	25.9 [23.5–29.3]	25.1 [21.7–28.3]	0.04	25.1 [22.0–29.0]	25.3 [22.1–28.5]	0.8
Pain evaluation (Visuo-Analogic Scale) (/100)	51 [32–91]	81 [50–100]	50 [22–88]	<0.001	52 [40–90]	51 [29–92]	0.8	65 [50–96]	51 [30–90]	0.1
SPPB (/12)‡	2 [1–4]	3 [1–5]	1 [0–4]	0.002	3 [1–6]	1 [0–4]	<0.001	3 [1–6]	2 [0–4]	0.003
MNA (714) ¥	11 [9–12]	11 [10–12]	10 [9–12]	<0.001	11 [10–12]	10 [9–12]	<0.001	11 [10–12]	11 [9–12]	0.006
CCI^+^ (/24)	6 [4–7]	5 [4–7]	6 [5–7]	0.1	6 [4–7]	6 [5–7]	0.6	6 [4–7]	6 [5–7]	0.7
AMTS (/10)•	7 [5–9]	7 [4.5–9]	8 [5–9]	0.1	8 [6–10]	7 [4–9]	<0.001	8 [6–10]	7 [5–9]	0.1
Comorbidities n (%)										
Heart failure	163 (30.3)	52 (25.7)	111 (33.0)	0.04	40 (28.7)	123 (30.8)	0.6	13 (25.0)	150 (30.8)	0.6
Hypertension	335 (62.0)	123 (60.5)	212 (62.9)	0.6	82 (58.9)	253 (63.0)	0.4	26 (50.0)	309 (63.3)	0.6
Diabetes Mellitus	85 (15.7)	30 (14.7)	55 (16.3)	0.6	25 (18.0)	60 (15.0)	0.4	8 (15.3)	77 (15.8)	0.9
Respiratory insufficiency	30 (5.5)	9 (4.4)	21 (6.2)	0.3	7 (5.0)	23 (5.7)	0.7	3 (5.7)	27 (5.5)	0.9
Respiratory insufficiency requiring O2	12 (2.2)	2 (1.0)	10 (2.9)	0.1	2 (1.4)	10 (2.5)	0.4	0 (0)	12 (2.4)	0.2
Peripheral Artery Disease	18 (3.3)	5 (2.4)	13 (3.9)	0.3	4 (2.9)	14 (3.5)	0.7	1 (1.9)	17 (3.5)	0.5
Hypothyroidism	67 (12.4)	20 (9.9)	47 (13.9)	0.1	15 (10.7)	52 (13.0)	0.1	6 (11.5)	61 (12.5)	0.009
Hyperthyroidism	10 (1.8)	1 (0.4)	9 (2.6)		0.03	3 (2.1)	7 (1.7)	0.7	0 (0)	10 (2.0)
Moderate/ severe CKD ii	141 (26.2)	53 (26.2)	88 (26.1)	0.7	40 (28.7)	101 (25.3)	0.1	17 (32.6)	124 (25.5)	0.5
Alzheimer’s Disease	133 (24.8)	63 (31.3)	70 (20.9)	0.02	28 (20.1)	105 (26.5)	0.08	11 (21.1)	122 (25.2)	0.5
Depression€	197 (36.6)	69 (33.9)	128 (38.2)	0.3	48 (34.5)	149 (37.3)	0.5	17 (32.6)	180 (37.0)	0.5
Chronic pain	228 (42.3)	66 (32.5)	162 (48.3)	0.001	46 (33.0)	182 (45.6)	0.02	12 (23.0)	216 (44.4)	0.01

‘Mental Vitality defined as a Score of 0, “Physical Vitality defined as HGS≥23Kg in male and ≥13.5 Kg in female, “‘Combined Vitality defined as presenting both mental and physical vitality, *GDS; Geriatric Depression Scale, °ADL; Activity of Daily Living, †IADL; Instrumental Activity if Daily Living, ***BMI; Body Mass Index, **Visuo-analogic scale (score 0–100; a score of 100 means no pain, a score of 0 means extreme pain), ‡SPPB; Short Physical Performance Battery, ¥MNA; Mini Nutritional Assessment test, ^+^CCI; Charlson Comorbidity Index,•AMTS; Abbreviated Mental Test Score, € Depression diagnosed by the physician, iiCKD; chronic Kidney Disease

### Evolution of ADL score

ADL scores significantly decreased after the 1-year follow-up among participants with low vitality and high vitality, according to all definitions (Table 2). Participants classified with high physical vitality presented a lower ADL decrease compared to participants with low physical vitality (mean between-group difference: −0.3, SE = 0.1; p = 0.02). On the other hand, people with high mental vitality showed a statistically significant higher decrease in ADL, compared to the low mental vitality individuals (mean between-group difference: 0.3, SE = 0.1; p = 0.02). No between-group differences were observed according to the combined definition of vitality.

**Table 2:** Evolution of the ADL score at 6 and 12 months among nursing home residents according to the three definitions of vitality (mental, physical and combined)

**Mental Vitality’**								
	**High vitality**	**Low vitality**	**High vitality**	**p**	**Low vitality**	**p**	**Between group Adjusted Mean Difference (Standard Error; p)**	**βCoefficient for time-by-Vitality Group interaction (95% CI; p)**^**a**^
**ADL**	**ADL Score (Standard Error)**		**Within Group Adjusted Mean Difference (Standard Error)**^**a, b**^					
T0	3.1 (0.1)	3.0 (0.1)						
T6	2.4 (0.1)	2.6 (0.1)	−0.7 (0.1)	<0.001	−0.4 (0.1)	<0.001	0.2 (0.1; 0.1)	− 0.2 (−0.5 to −0.01; 0.04)
T12	2.3 (0.1)	2.6 (0.1)	−0.8 (0.1)	<0.001	−0.4 (0.1)	<0.001	0.3 (0.1; 0.02)	− 0.3 (−0.6 to −0.1; 0.006)
**Physical Vitality”**								
	**High vitality**	**Low vitality**	**High vitality**	**p**	**Low vitality**	**p**	**Between group Adjusted Mean Difference (Standard Error; p)**	**β Coefficient for time-by-Vitality Group interaction (95% CI; p)**^**a**^
**ADL**	**ADL Score (Standard Error)**		**Within Group Adjusted Mean Difference (Standard Error)**^**a, b**^					
T0	3.2 (0.1)	3.0 (0.1)						
T6	2.7 (0.1)	2.4 (0.1)	−0.5 (0.1)	<0.001	−0.5 (0.1)	<0.001	−0.2 (0.1; 0.1)	− 0.001 (−0.2 to 0.2; 0.9)
T12	2.7 (0.1)	2.4 (0.1)	−0.5 (0.1)	<0.001	−0.6 (0.1)	<0.001	−0.3 (0.1; 0.02)	0.1 (−0.1 to 0.4; 0.4)
**Combined Vitality’”**								
	**High vitality**	**Low vitality**	**High vitality**	**p**	**Low vitality**	**p**	**Between group Adjusted Mean Difference (Standard Error; p)**	**βCoefficient for time-by-Vitality Groupinteraction (95% CI; p)**
**ADL**	**ADL Score (Standard Error)**		**Within Group Adjusted Mean Difference (Standard Error)**^**a, b**^					
T0	3.1 (0.2)	3.0 (0.1)						
T6	2.5 (0.2)	2.5 (0.1)	−0.5 (0.2)	0.004	−0.5 (0.1)	<0.001	0.02 (0.2; 0.9)	−0.01 (−0.4 to 0.4; 0.9)
T12	2.5 (0.2)	2.5 (0.01)	−0.6 (0.2)	0.002	−0.5 (0.1)	<0.001	0.02 (0.2; 0.9)	0.06 (−0.5 to 0.3; 0.7)

a. Negative values indicates decrease in the ADL score compared to ADL at T0; b. The ADL score at T0 was the reference value; Analysis adjusted for age, sex, duration of institutionalization, IADL, SPPB, MNA and Charlson Comorbidity Index at inclusion, post 6 months and post 12 months; ‘Mental Vitality defined as a Score of 0 based on three questions from the Geriatric Depression Scale, “Physical Vitality defined as HGS≥23Kg in male and ≥13.5 Kg in female (4th quartile), “‘Combined Vitality defined as presenting both Mental and Physical vitality, °ADL; Activity of Daily Living; (Mental Vital group: 204, Mental Low Vitality group: 337) patients were entered into the regression model (adjusted for age, sex, duration of institutionalization, IADL, SPPB, MNA and Charlson Comorbidity Index)

A sensitivity analysis was performed by removing subjects with dementia and/or depression (n=279) (Table S1). The non-demented and/or depressed residents with very low vitality had a significant decline in ADL after 1 year, while participants with very high vitality did not. However, mean between-group difference in the evolution of the ADL score between very high and very low vitality was not statistically significant.

### Hospitalization

Of the 511 individuals with data on vitality and hospitalization, 180 were hospitalized during the follow-up. For the combined vitality definition, Cox regression analysis showed a reduction in the risk of hospitalization in individuals with very vitality, compared to very low vitality, after adjustments (Table 3). No differences in hospitalization between vitality groups were found in separate models for physical and mental vitality after adjusting for confounders.

**Table 3:** Incidence of mortality and hospitalization according to each definition of vitality

**Model**	**Mental Vitality’**		**Physical Vitality”**		**Combined Vitality’”**	
	**HR [95% CI]**	**p**	**HR [95% CI]**	**p**	**HR [95% CI]**	**p**
Mortality (adjusted for age and sex) n=532, 89 events	1.1 [0.7,1.7]	0.5	0.2 [0.1,0.5]	0.001	0.6 [0.2,1.6]	0.3
Mortality (adjusted for age, sex, duration of institutionalization, IADL, SPPB, MNA, CCI and AMTS) n=416, 89 events	1.2 [0.7,2.1]	0.3	0.3 [0.1,0.7]	0.009	0.6 [0.2,1.8]	0.4
First hospitalization (adjusted for age and sex) n=511, 180 events	0.7 [0.5,0.9]	0.004	0.7 [0.4,1.0]	0.051	0.5 [0.2,0.9]	0.03
First hospitalization (adjusted for age, sex, duration of institutionalization, IADL, SPPB, MNA, CCI and AMTS) n=416, 180 events	0.7 [0.5,1.0]	0.09	0.7 [0.4,1.0]	0.1	0.4 [0.2,0.9]	0.038

Cox regression analysis adjusted for age and sex then age, sex duration of institutionalization, IADL, SPPB, MNA, CCI and AMTS, displaying the associations of mental, physical and total vitality with mortality and first hospitalization; ‘ental Vitality defined as a Score of 0, “Physical Vitality defined as HGS≥23Kg in male and ≥13.5 Kg in female, “‘Combined Vitality defined as presenting both Mental and Physical vitality; HR, hazard ratio; CI, confidence interval; IADL, Instrumental Activity if Daily Living; SPPB, Short Physical Performance Battery; MNA, Mini Nutritional Assessment test; CCI, Charlson Comorbidity Index; AMTS, Abbreviated Mental Test Score.

### Mortality

Of the 532 individuals with data on vitality and death, 89 events were recorded. Residents with high physical vitality had a significantly lower mortality rate (table 3), even after adjustments. No other associations were found for mortality risk between vital and non-vital subjects according to both mental and combined vitality definitions.

## Discussion

This study shows that NH residents with very high vitality using a definition that combines HGS (physical vitality) and three questions from the GDS (mental vitality) was associated with a decreased risk of hospitalization. Moreover, individuals classified as high physical vitality were associated with a decreased risk of mortality and a lower decrease of ADL score. However, high mental vitality was associated with a worsening of the ADL score.

To our knowledge, the objective measurement of vitality per se has never been studied in this population. In our study, NH residents with very high vitality had a decreased risk of hospitalization, compared to individuals with very low vitality. Consequently, poor HGS is a surrogate marker for adverse events and has been shown to be related with a higher risk of hospitalization (21). Also, in the same cohort of our study, lack of energy (measured with one of the questions of the GDS scale) was shown to be associated with a higher risk of hospitalization (22). As such, combining these two measurements could be a potential screening tool to predict adverse events in this population. Further studies are needed to confirm this hypothesis.

In our sample, participants with high physical vitality presented lower ADL score decline over time. Furthermore, among a subsample of non-demented individuals, very high vitality was associated with better ADL score evolution, compared to individuals with very low vitality. These results are in accordance with a study that showed that community-dwelling older adults with low vitality has been associated with higher risk of frailty and lower IADL scores (23). More interestingly, residents with high mental vitality had significantly worsened ADL scores compared low vitality, but such result was not observed among a subsample of non-demented and non-depressed individuals. This indicates that mental vitality measured with 3 items from the GDS may not be adequate for this population. Penninx and colleagues (24) found that lower emotional vitality, which included anxiety, depressive symptoms and happiness was related to subsequent new disability and mortality in disabled older women. That study suggests that a broader definition of mental vitality is probably needed in order to improve its predictive value in NH residents with various cognitive disorders.

Moreover, people with high physical vitality had a significantly lower mortality risk compared to individuals with low physical vitality. This result is not surprising because it has been widely demonstrated that HGS is also a predictor of mortality (25, 26). As far as mental and combined vitality is concerned, the risk of mortality between high vitality and low vitality were statistically similar. More studies with longer follow-ups are needed to assess if vitality defined by the combination of physical measures and psychology-related subjective items can be considered as a potential predictor of mortality in the NH setting.

To the best of our knowledge, this is the first study that has operationalized a definition of vitality that covers both a psychological aspect and an objectively measured physical assessment in the NH setting, while previous studies focused mainly on one of the two aspects only (27–29). Other strengths of our study are the prospective design and the relatively large sample size of NH residents; even though the 1-year follow-up is a short time length for most populations, in very old and vulnerable people, such as those living in NH, significant and clinically meaningful declines were observed (eg, almost 20% of the population died). Limitations are also worth mentioning: NH residents are particularly more at risk of adverse events, which means the results of this study are not generalizable to community-dwelling older adults; we used a within-study cut-off for HGS due to the lack of a well-established clinical cut-off for this measurement in NH subjects. NH residents had lower ADL scores already at baseline, rendering it difficult to observe a decline in ADLs (30) due to a potential floor effect.

## Conclusions and Implications

In conclusion, this is the first study investigated the predictive value of three potential definitions of vitality among the institutionalized elderly population. Very high vitality combining physical and psychological elements was associated with a lower risk of hospitalization, whereas HGS (physical vitality) was associated with a reduced risk of mortality. High mental vitality was associated with worsening of ADL in the total sample, but not in the subsample of non-demented subjects, suggesting this vitality definition, when employed alone, may be not appropriate for the very old and vulnerable population living in NH. Larger studies, with longer observation periods and using different measurements of mental vitality are needed to determine the best operational definition of vitality in this population.

## Electronic supplementary material

Table S1. Sensitivity analysis of ADL score variation over time in vitality for nursing home residents without dementia.

## Notes

*Ethical Standards:* The INCUR study followed the principles of the Delcaration of Helsinki and the ethical standards complied. The study protocol was approved by the Ethics Committee of the Centre Hospitalier de Toulouse and the Consultative Committee for the Treatment of Research Information on Health.
