# Supplementary material for: Defining Vitality Using Physical and Mental Well-Being Measures in Nursing Homes: A Prospective Study
Source: J Nutr Health Aging. 2019 Oct 19;24(1):37–42. doi: 10.1007/s12603-019-1285-8 (PMC6934632; doi:10.1007/s12603-019-1285-8)
Supplement: Supplementary file 1 — Table S1. Sensitivity analysis of ADL score variation over time in vitality for nursing home residents without dementia. [file 12603_2019_1285_MOESM1_ESM.docx]

|  | | Mental Vitality′ | | | | | | | |
| --- | --- | --- | --- | --- | --- | --- | --- | --- | --- |
|  | High vitality | Low vitality | High vitality | p | Low vitality | p | Between group Adjusted Mean Difference (SE; p) | β Coefficient for time-by-Vitality Group interaction (95% CI; p) |  |
| ADL | ADL Score  (Standard Error) | | Within Group Adjusted Mean Difference  (Standard Error)^a,b^ | | | |  |  |  |
| T0 | 3.3 (0.1) | 3.2 (0.1) |  | | | |  |  |  |
| T6 | 2.6 (0.1) | 2.8 (0.1) | -0.6 (0.1) | **<0.001** | -0.4 (0.1) | **<0.001** | 0.15 (0.2; 0.4) | - 0.2 (-0.5 to 0.1; 0.3) |  |
| T12 | 2.5 (0.1) | 2.8 (0.1) | -0.7 (0.1) | **<0.001** | -0.4 (0.1) | **<0.001** | 0.2 (0.2;0.2) | - 0.2 (-0.6 to -0.1; 0.1) |  |
|  | | Physical Vitality″ | | | | | | | |
|  | High vitality | Low vitality | High vitality | p | Low vitality | p | Between group Adjusted Mean Difference (SE; p) | β Coefficient for time-by-Vitality Group interaction (95% CI; p) |  |
| ADL | ADL Score  (Standard Error) | | Within Group Adjusted Mean Difference  (Standard Error)^a,b^ | | | |  |  |  |
| T0 | 3.3 (0.1) | 3.2 (0.1) |  | | | |  |  |  |
| T6 | 2.7 (0.1) | 2.7 (0.1) | -0.5 (0.1) | **0.001** | -0.5 (0.1) | **<0.001** | -0.04 (0.2; 0.2) | - 0.06 (-0.4 to 0.3; 0.7) |  |
| T12 | 2.8 (0.1) | 2.6 (0.1) | -0.5 (0.1) | **0.004** | -0.6 (0.1) | **<0.001** | -0.2 (0.2; 0.3) | 0.1 (-0.2 to 0.5; 0.5) |  |
|  | | Combined Vitality‴ | | | | | | | |
|  | High vitality | Low vitality | High vitality | p | Low vitality | p | Between group Adjusted Mean Difference (SE; p) | β Coefficient for time-by-Vitality Group interaction (95% CI; p) |  |
| ADL | ADL Score  (Standard Error) | | Within Group Adjusted Mean Difference  (Standard Error)^a,b^ | | | |  |  |  |
| T0 | 3.0 (0.2) | 3.3 (0.1) |  | | | |  |  |  |
| T6 | 2.5 (0.2) | 2.7 (0.1) | -0.4 (0.2) | 0.05 | -0.5 (0.1) | **<0.001** | 0.2 (0.3; 0.4) | 0.07 (-0.4 to 0.6; 0.7) |  |
| T12 | 2.5 (0.2) | 2.7 (0.1) | -0.4 (0.2) | 0.07 | -0.6(0.1) | **<0.001** | 0.1 (0.3; 0.5) | 0.1 (-0.4 to 0.6; 0.6) |  |

**Table S1. Sensitivity analysis of ADL score variation over time in vitality for nursing home residents without dementia.**

^a^Negative values indicates decrease in the ADL score compared to the ADL at T0

^b^ The ADL score at T0 was the reference value

Group adjusted for age, sex, duration of institutionalization, IADL, SPPB, MNA and Charlson Comorbidity Index at inclusion, post 6 months and post 12 months.

′Mental Vitality defined as a Score of 0 based on three questions from the Geriatric Depression Scale, ″Physical Vitality defined as HGS≥23Kg in male and ≥13.5 Kg in female, ‴Combined Vitality defined as presenting both Mental and Physical vitality, ^◦^ADL; Activity of Daily Living,

Sensitivity analysis removing patients diagnosed by the physician with dementia and depression: 262 NHRs in the sample population with median AMTS median score of 8 [5-10] and a GDS median score of 2 [1-4].

(Mental Vital group: 98, Mental Non Vital group: 164) patients were entered into the regression model (adjusted for age, sex, duration of institutionalization, IADL, SPPB, MNA and Charlson Comorbidity Index)

(Physical Vital group: 75, Physical Non Vital group: 187) patients were entered into the regression model (adjusted for age, sex, duration of institutionalization, IADL, SPPB, MNA and Charlson Comorbidity Index)

(Total Vital group: 30, Mental Non Vital group: 232) patients were entered into the regression model (adjusted for age, sex, duration of institutionalization, IADL, SPPB, MNA and Charlson Comorbidity Index)
